# Metasurfaced Reverberation Chamber

**DOI:** 10.1038/s41598-018-20066-0

**Published:** 2018-01-25

**Authors:** Hengyi Sun, Zhuo Li, Changqing Gu, Qian Xu, Xinlei Chen, Yunhe Sun, Shengchen Lu, Ferran Martin

**Affiliations:** 1 Key Laboratory of Radar Imaging and Microwave Photonics, Ministry of Education, College of Electronic and Information Engineering, Nanjing University of Aeronautics and Astronautics, Nanjing, 211106 China; 2 CIMITEC, Departament d’Enginyeria Electrònica, Universitat Autònoma de Barcelona, 08193 Bellaterra Barcelona, Spain

## Abstract

The concept of metasurfaced reverberation chamber (RC) is introduced in this paper. It is shown that by coating the chamber wall with a rotating 1-bit random coding metasurface, it is possible to enlarge the test zone of the RC while maintaining the field uniformity as good as that in a traditional RC with mechanical stirrers. A 1-bit random coding diffusion metasurface is designed to obtain all-direction backscattering under normal incidence. Three specific cases are studied for comparisons, including a (traditional) mechanical stirrer RC, a mechanical stirrer RC with a fixed diffusion metasurface, and a RC with a rotating diffusion metasurface. Simulation results show that the compact rotating diffusion metasurface can act as a stirrer with good stirring efficiency. By using such rotating diffusion metasurface, the test region of the RC can be greatly extended.

## Introduction

Metamaterials constitute one of the most significant and interesting topics in electromagnetic field theory. Planar metamaterials, particularly those operating in the optical domain, can be readily fabricated by means of currently available technologies, such as lithography or nano-printing methods. Such technologies allow for the implementation of single-layer or multilayer stacks of planar metamaterial structures. Planar metamaterials made by a two-dimensional (2D) array of “meta-atoms” are called metasurfaces, which can be considered to be the 2D equivalent of bulk metamaterials^1–3^. Cui *et al*. proposed the concept of a coding metasurface in 2014^4^, which is well suited to a variety of applications, such as the control of the radiation beams of antennas, the reduction of the scattering features of targets, and the fabrication of smart metamaterials. Diffusion coding metasurfaces have recently been proposed for radar cross section (RCS) reduction^5–7^. The concept of coding metasurface can also be applied in the area of electromagnetic compatibility (EMC), particularly in producing diffusive back scattering in reverberation chambers (RCs), which are well-known facilities to carry out EMC tests^8–12^. RCs, typically implemented by rectangular cavities, use mode stirring or mode tuning technology to change the boundary conditions of the electromagnetic fields, in order to produce a statistically uniform field distribution^13–16^. In^13^, the field uniformity is specified as a standard deviation from the mean value of the normalized maximum values obtained at each of eight locations during one rotation of the stirrer. Currently, most standard RCs are equipped with one or two mechanical stirrers in order to achieve a uniform field distribution. However, such stirrers occupy a large volume in the RC. To overcome the drawback of the size and maintenance of stirrers, the use of diffusers to enhance the scattering in the RC is proposed (note that diffusers are much smaller than stirrers)^17–25^. Many types of diffusers exist, including the Schroeder quadratic residue diffuser (QRD), the maximum length diffuser (MLD) and the primitive root residue diffuser (PRD). All types of diffusers have a specific design that depends on a pseudorandom sequence. Based on the mathematical group of Galois fields, a design rule for all types of diffusers can be developed^26–28^. However, these diffusers consist of rectangular metal blocks with different heights and have narrow operating bandwidths. Combining the concept of coding metasurface with the implementation mechanism of diffusers, a random coding diffusion metasurface with ultra-thin thickness (and hence with a smaller volume than other diffusers) and wide operation bandwidth, can be implemented.

In this work, for the metasurfaced RC, we first design a 1-bit random coding metasurface by employing a genetic algorithm that can provide an optimized distribution of unit cells in order to achieve all-direction backscattering. Then, simulations are performed to investigate the scattering characteristic of the 1-bit random coding metasurface in free space. The field uniformity in a mechanical stirrer RC and that in the same RC (i.e., with mechanical stirrer) loaded with a designed (fixed) metasurface is compared in detail. Finally, we consider a RC in which the mechanical stirrer is removed and the metasurface is rotated 360° in equally discrete steps (metasurfaced RC). Simulation results show that the conventional RC with rotating 1-bit random coding diffusion metasurface and without mechanical stirrer can effectively enlarge the test zone of the RC and obtain the field uniformity at least the same level as that in a traditional mechanical stirrer RC.

## Results

### Unit cell properties and the design of a diffusion metasurface

**Figure 1:**
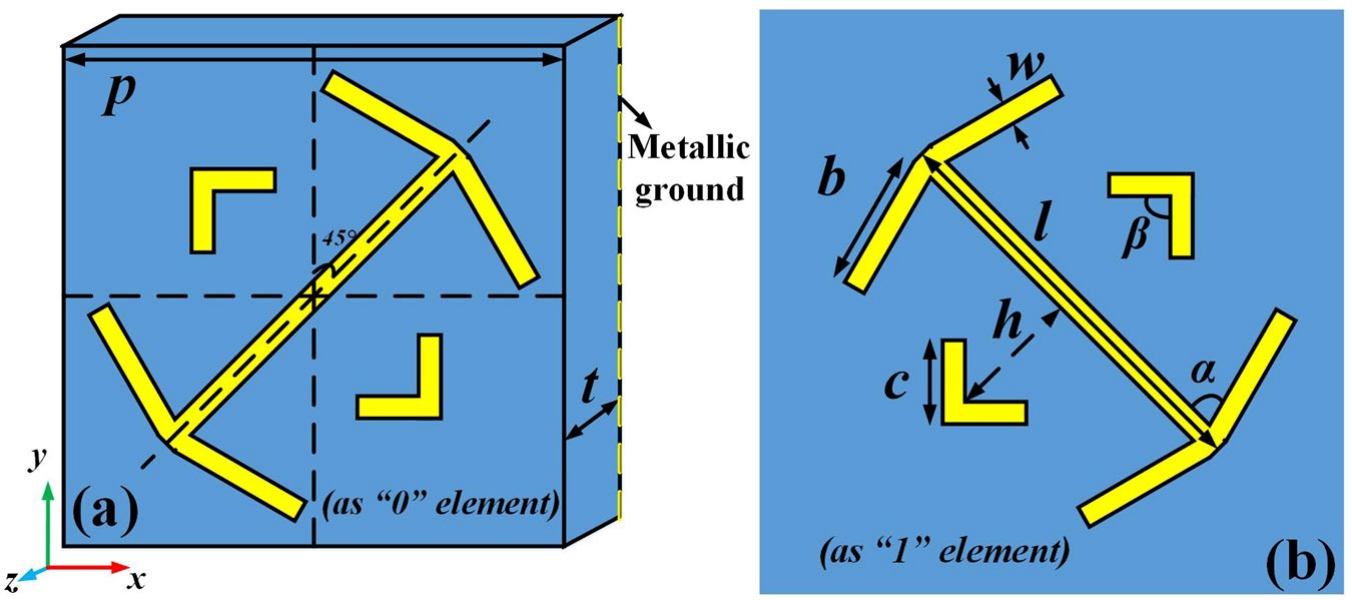
Front views of “0” and “1” element unit cells. The unit cell structure with an angle of 45° to the y-axis represents the “0” element (**a**), and the structure rotated 90° counter-clockwise about the z-axis is the “1” element (**b**).

The multilayer structure for the reflection diffusion metasurface consists of a top metallic structure, an intermediate dielectric layer, and a bottom metallic ground layer. The metasurface, constructed by a periodic or non-periodic array of unit cells with asymmetric structures, can be viewed as an anisotropic homogeneous material with dispersive permittivity and permeability. Generally, arbitrary asymmetric geometry leads to different phase responses under normal incidence. When a plane wave impinges upon the top metallic layer of the metasurface, both reflected and transmitted waves are generated. The transmitted waves undergo multiple reflections between the top and bottom metallic layers, and they mutually interfere to create the final reflected wave. The dielectric spacer and metallic ground increase the design degrees of freedom for controlling the amplitude and phase of the waves produced in the multiple reflections. Therefore, we can increase the bandwidth of the anisotropic reflection from the three-layer structure by optimizing the dielectric spacer. Based on these principles, and to concisely demonstrate our method and facilitate the simulation procedure, we choose the structure that we used for the design of a metasurface intended for radar cross section (RCS) reduction as a candidate (see Fig. 1^5^), which has an ultra-wide band and broad-angle characteristic. By choosing the thickness of the dielectric spacer as a design parameter, we studied the unit cell characteristics using interference theory, as presented in^29,30^. As a coupled system, all possible near-field interactions between the top metallic structure and the metallic ground sheet can be accurately taken into account. By optimizing the structure parameters of the unit cells in the diffusion metasurface, we obtained the geometrical dimensions shown in Fig. 1(a,b), i.e., ***l*** = 43.64 mm, ***b*** = 15.59 mm, ***c*** = 8.97 mm, ***h*** = 16 mm, ***α*** = 80°, ***β*** = 90°, ***w*** = 4 mm, ***p*** = 60 mm, ***t*** = 31 mm. The thickness of the metallic layer is 0.035 mm, and the dielectric layer is the *Rogers RT 5880* with the dielectric constant ***ε***_***r***_ = 2.2 and the loss tangent tan***δ*** = 0.0009.

**Figure 2:**
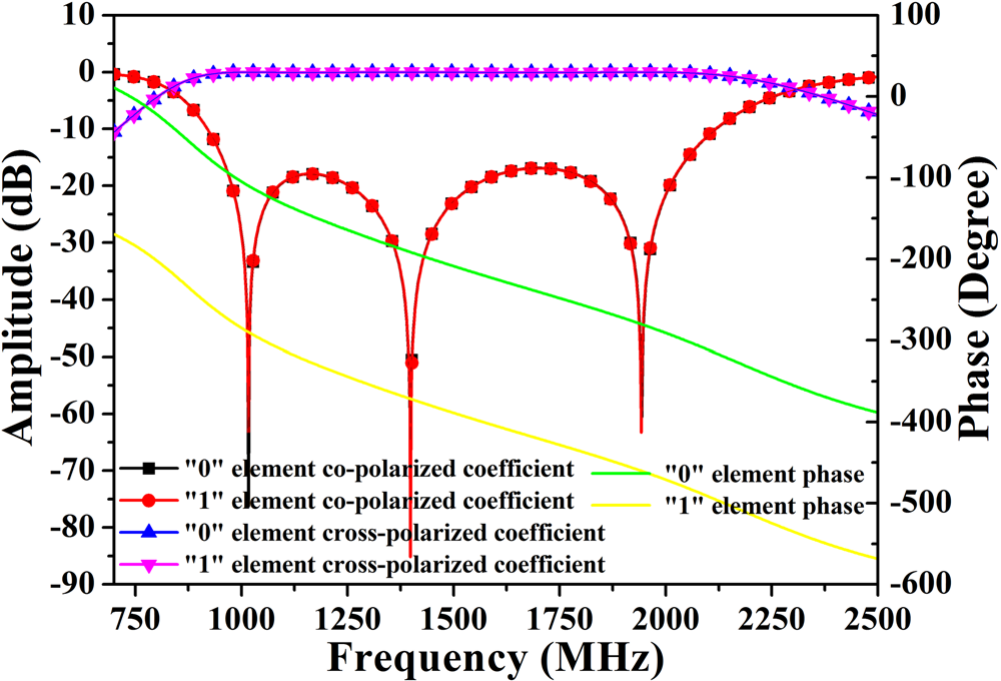
The reflected amplitudes and phases of the “0” and “1” elements under normal incident waves in simulation. Because of the anisotropy of the metasurface, there is a phase difference (∆φ) between the “0” and “1” elements.

In general, the unit cells of a diffusion metasurface exhibit different phase responses under normal incidence. The unit cells of the 1-bit coding metasurface with relative phase responses of π can be randomly arranged in the metasurface to achieve all-direction backscattering. Fig. 2 depicts the reflection characteristics of the structure calculated by *CST Microwave Studio*. Owing to the full metallic ground condition on the backside of the device, the entire structure perfectly reflects the incident waves (assuming that losses are not present). To satisfy the periodic boundary in the element simulation, a lattice containing 4 × 4 anisotropic elements in the same orientation is generated. Thus, different coding sequences can be translated into an orientation of lattices. Once the lattice has been prepared, we can determine the optimal layout of the 1-bit random coding diffusion metasurface.

**Figure 3:**
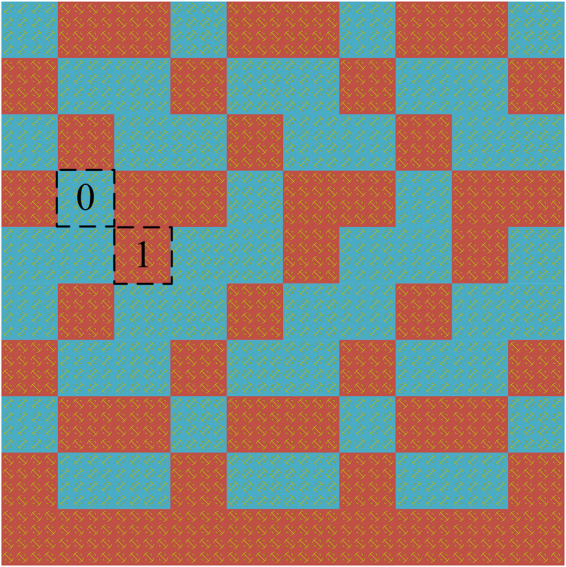
The 1-bit random coding diffusion metasurface distributions on a large area (2.4 × 2.4 m^2^) containing 100 lattices. The “0” and “1” lattices comprise 4 × 4 equivalent elements and are distinguished by blue and red colours, respectively.

**Figure 4:**
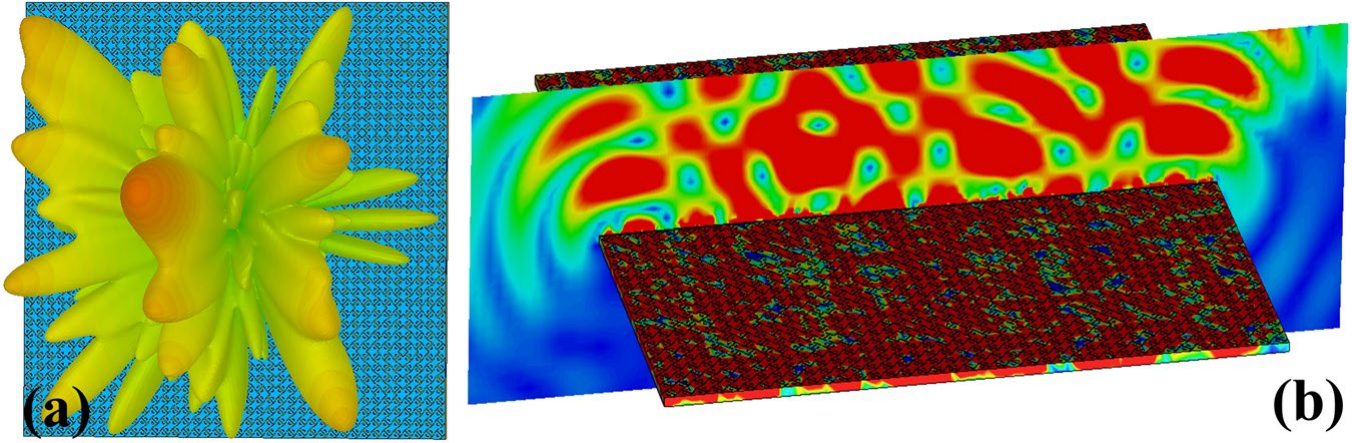
Simulated far-field and near-field pattern of the metasurface under normal incidence at 1 GHz. (**a**) The far-field scattering pattern. (**b**) The surface current distribution and the near-electric-field distributions of the metasurface.

To obtain all-direction backscattering and thus achieve uniform field distribution under normal incidence, an efficient genetic algorithm that allows us to find the optimal arrangement of coding elements is employed. During the optimization, we use a far-field pattern prediction algorithm^5^ as an auxiliary module to reduce the effort for large-scale full-wave simulations. For instance, in the RC, we consider a metasurface with a large area of 2.4 m × 2.4 m (8*λ* × 8*λ* at 1 GHz), which contains 10 × 10 = 100 lattices. After several optimization iterations, the arrangement of the 1-bit random coding diffusion metasurface is determined, as illustrated in Fig. 3. The metasurface generates a diffuse far-field scattering pattern, as depicted in Fig. 4(a). The pattern obtained by full-wave simulation is in agreement with the result of the optimization algorithm. Fig. 4(b) shows the surface current distribution and the near-electric-field distributions of the metasurface under normal incidence at 1 GHz. The figure demonstrates that the elements on the metasurface present different resonant states, which is critical to disturbing the equiphase reflection.

### Theoretical analysis and simulations of the RC

**Figure 5:**
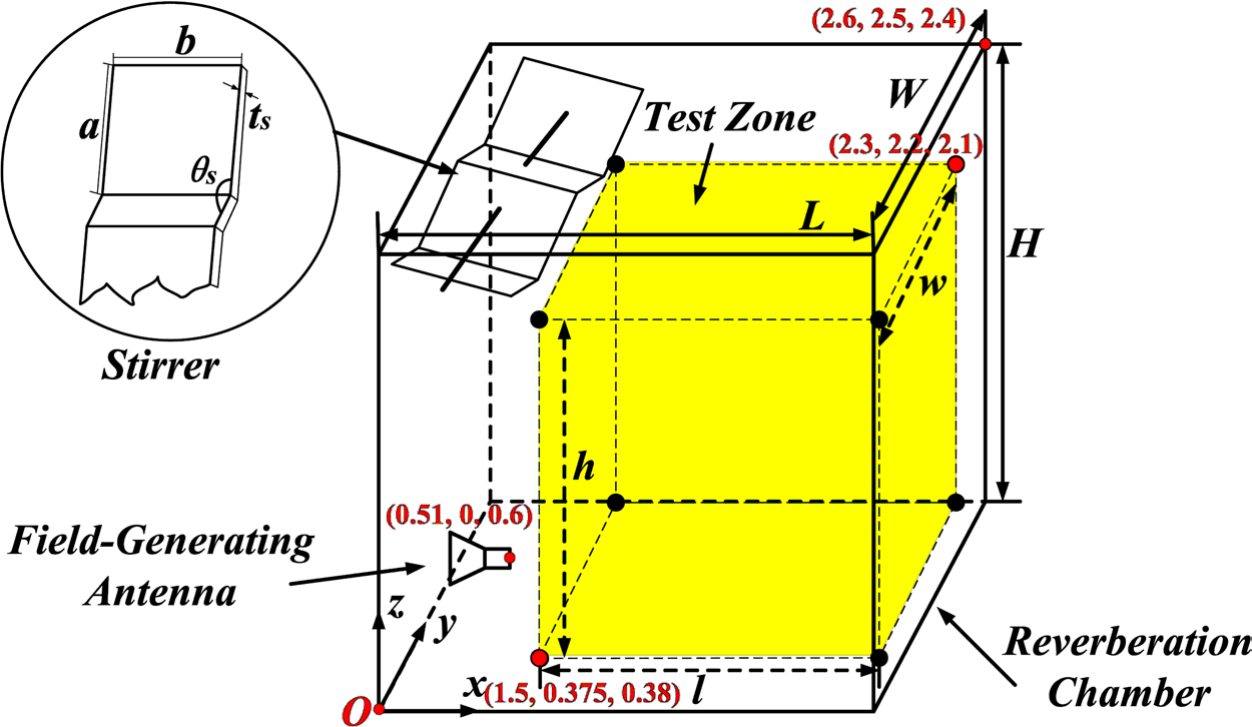
The schematic diagram of the mechanical stirrer RC. The field-generating antenna is arranged in the centre of the RC, and the yellow volume is the test zone (***l*** × ***w*** × ***h*** = 0.8 m × 1.825 m × 1.72 m). The stirrer is made of four metallic sheets with ***a*** = 800 mm and ***b*** = 900 mm, the angle between two sheet layers is ***θ***_***s***_ = 120° and the thickness of the stirrer is ***t***_***s***_ = 5 mm.

Usually, the cavity mode in the RC is a particular field distribution generated by standing waves. The modes in a cavity are governed by the boundary conditions. For an ideal, lossless, empty, closed rectangular cavity (as shown in Fig. 5) of dimensions ***L*** (length), ***W*** (width) and ***H*** (height), the resonance frequency ***f***_***l***,***m***,***n***_ of a certain mode is given by.

1 $${{\boldsymbol{f}}}_{{\boldsymbol{l}},{\boldsymbol{m}},{\boldsymbol{n}}}=\frac{{\rm{1}}}{{\rm{2}}\sqrt{{\boldsymbol{\mu }}{\boldsymbol{\varepsilon }}}}\sqrt{{(\frac{{\boldsymbol{l}}}{{\boldsymbol{L}}})}^{2}+{(\frac{{\boldsymbol{m}}}{{\boldsymbol{W}}})}^{2}+{(\frac{{\boldsymbol{n}}}{{\boldsymbol{H}}})}^{2}},$$

where ***ε*** and ***μ*** are the permittivity and permeability of the medium inside the cavity, respectively, and ***l***, ***m***, ***n*** are the mode indices (at least two of which are nonzero). In our simulations, the medium in the cavity RC is air. The total number of the resonant modes ***N***(***f***), being an important factor that characterizes the mode distribution and the field uniformity in a RC^22,31^, can be calculated as

2 $${\boldsymbol{N}}({\boldsymbol{f}})=\frac{8{\boldsymbol{\pi }}}{3}{\boldsymbol{LWH}}{(\frac{{\boldsymbol{f}}}{{\boldsymbol{c}}})}^{{\rm{3}}}-({\boldsymbol{L}}+{\boldsymbol{W}}+{\boldsymbol{H}})\frac{{\boldsymbol{f}}}{{\boldsymbol{c}}}+\frac{1}{2},$$

in which ***f*** is the operating frequency and ***c*** is the speed of light in free space, approximately 3 × 10^8^ m/s. Thus, the mode density can be obtained by

3 $$\frac{{\boldsymbol{dN}}({\boldsymbol{f}})}{{\boldsymbol{df}}}=\frac{8{\boldsymbol{\pi }}{\boldsymbol{LWH}}}{{\boldsymbol{c}}}{(\frac{{\boldsymbol{f}}}{{\boldsymbol{c}}})}^{2}-\frac{{\boldsymbol{L}}+{\boldsymbol{W}}+{\boldsymbol{H}}}{{\boldsymbol{c}}},$$

Note that in actual structures, losses can become relevant because they will reduce the *Q* factor of the cavity and hence will alter the calculations in equations (2) and (3). In this work, to meet the standard requirements, we set the dimensions of ***L***, ***W*** and ***H*** as 2.6 m, 2.5 m and 2.4 m, respectively. Thus, there are 36215 modes in the frequency range from 800 MHz to 2000 MHz, and the mode density is from 9.26 to 58.06 modes/MHz. These results indicate that the operation spectrum we choose is overmoded and conforms to the standard test requirement.The test zone is bounded by eight isotropic field probes set at least ***λ***_***max***_**/4** (***λ***_***max***_** = *c*/*f***) from the metallic wall in the RC.

Owing to the field uniformity requirements in a RC, we rotate the mechanical stirrer 360° in equally sized discrete steps. The coordinate of the antenna, in meters, is (0.51, 0, 0.6) and the polarization is horizontal. The field uniformity is specified as a standard deviation from the normalized mean value of the normalized maximum values obtained at each of the eight locations during one rotation of the stirrer. The standard deviation is calculated using data from each probe axis independently and the total data set. The electric field distribution in the mechanical stirrer RC and the standard deviation of the probes are obtained from simulations. For a complete testing process, the standard deviation is expressed relative to the mean value and converted to dB as follows^14,18^. For electric fields in directions ***x***-, ***y***- and ***z***, i.e., ***E***_***x***_, ***E***_***y***_, ***E***_***z***_ the standard deviation is given by:

4 $${{\boldsymbol{\sigma }}}_{{\boldsymbol{i}}}=\sqrt{\frac{\sum _{{\boldsymbol{j}}=1}^{8}{({\overleftrightarrow{{\boldsymbol{E}}}}_{{\boldsymbol{i}},{\boldsymbol{j}}}-{\langle {\overleftrightarrow{{\boldsymbol{E}}}}_{{\boldsymbol{i}}}\rangle }_{8})}^{2}}{8-1}},$$

where ${\overleftrightarrow{{\boldsymbol{E}}}}_{{\boldsymbol{i}},j}$ is the individual normalized maximum E-field in the ***i***-direction (***i*** = ***x***, ***y***, ***z***) at the location ***j*** (with ***j*** = 1, 2 … 8), and ${\langle {\overleftrightarrow{{\boldsymbol{E}}}}_{i}\rangle }_{8}$ is the arithmetic mean of the normalized maximum field ${E}_{i}$ for all eight locations. For all vectors (***E***_***xyz***_):

5 $${{\boldsymbol{\sigma }}}_{{\boldsymbol{xyz}}}=\sqrt{\frac{{\sum _{{\boldsymbol{i}}=1}^{{\rm{3}}}\sum _{{\boldsymbol{j}}=1}^{{\rm{8}}}[{\overleftrightarrow{{\boldsymbol{E}}}}_{{\boldsymbol{i}},{\boldsymbol{j}}}-{\langle \overleftrightarrow{{\boldsymbol{E}}}\rangle }_{24}]}^{2}}{24-1},}$$

where the summation in the root extends to the three directions (plus the eight locations), ${\langle \overleftrightarrow{{\boldsymbol{E}}}\rangle }_{24}$ is the arithmetic mean of the normalized maximum field vectors corresponding to the three directions and eight locations, and $\langle {\overleftrightarrow{{\boldsymbol{E}}}}_{x,y,z}\rangle$ denotes the average of the normalized values across the probe positions for each probe axis of the E-field probe. In dB, the standard deviation, (4) or (5), is:

6 $${\boldsymbol{\sigma }}({\boldsymbol{dB}})=20{\mathrm{log}}_{10}(\frac{{\boldsymbol{\sigma }}+\langle {\overleftrightarrow{{\boldsymbol{E}}}}_{{\boldsymbol{x}},{\boldsymbol{y}},{\boldsymbol{z}}}\rangle }{\langle {\overleftrightarrow{{\boldsymbol{E}}}}_{{\boldsymbol{x}},{\boldsymbol{y}},{\boldsymbol{z}}}\rangle }),$$

where ***σ*** in the right-hand side is either ***σ***_***i***_ or ***σ***_***xyz***_.

**Figure 6:**
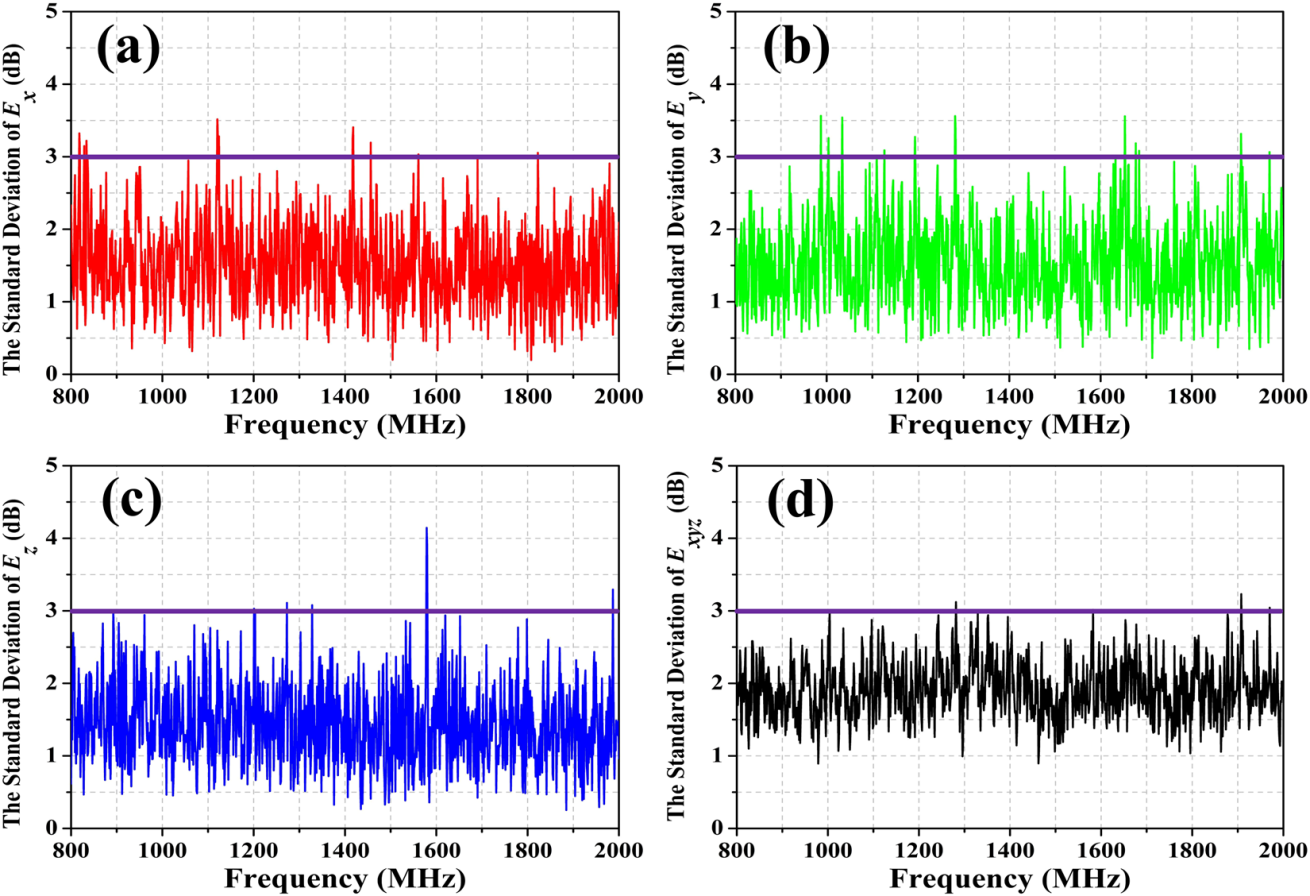
The standard deviations of the field strengths in the mechanical stirrer RC. The purple lines are the tolerance requirements for the standard deviation of;^13^ (**a**) the x-component, (**b**) the y-component, (**c**) the z-component, (**d**) the combination of xyz components.

The results of the standard deviation, obtained over one complete stirrer rotation with 12 stirrer steps, for the whole frequency regime are shown in Fig. 6. The whole structure (RC with mechanical stirrers and antenna) has been simulated in *CST Microwave Studio*. The conductivity of the conducting wall of the RC and the mechanical stirrer is set to ***σ***_***m***_ = 5.96 × 10^7^ S/m. In the figure, the acceptable limits for field uniformity decreed in^13^ are depicted as a purple solid line. According to^13^, the acceptable limits are as follows: the standard deviation should be within 4 dB below 100 MHz, decreasing linearly to 3 dB at 400 MHz, and within 3 dB above 400 MHz.

**Figure 7:**
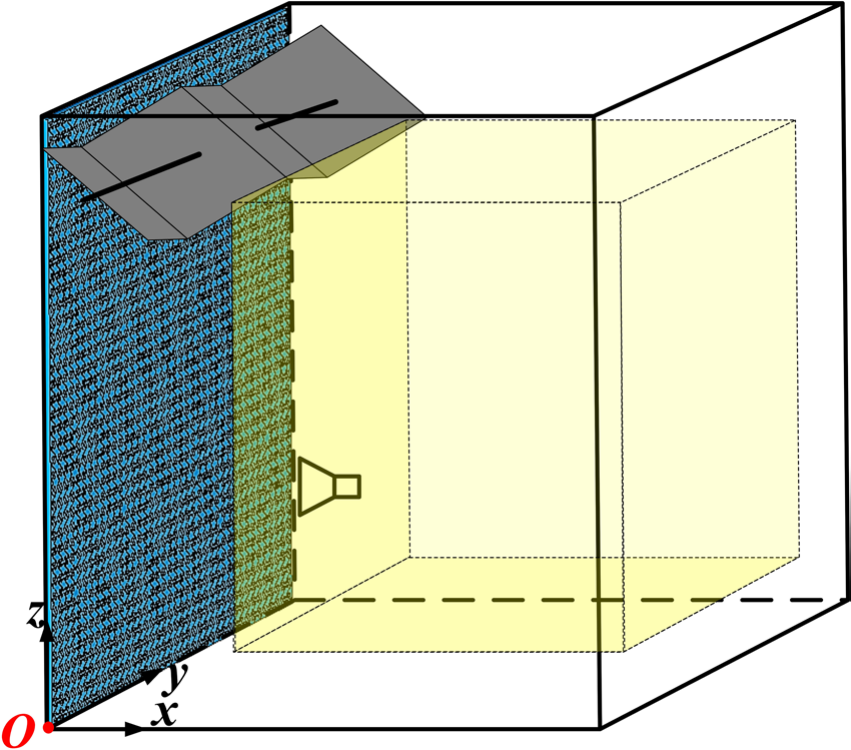
Schematic diagram of the 1-bit random coding diffusion metasurface loaded in the mechanical RC.

**Figure 8:**
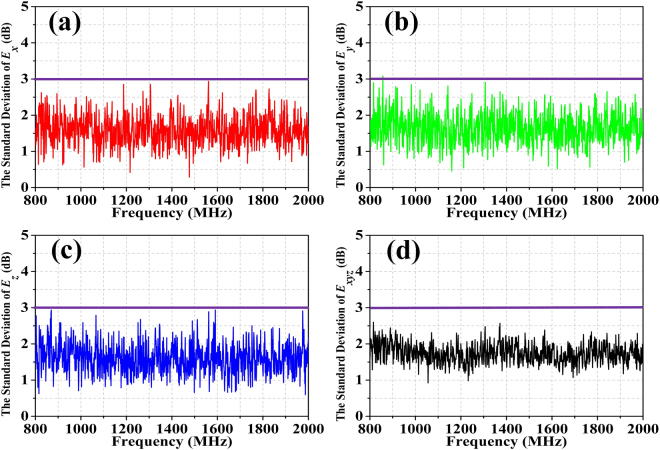
The standard deviations for the field uniformity of the test zone that is loaded with the fixed 1-bit random coding diffusion metasurface in the mechanical RC. The purple lines are the tolerance requirements for the standard deviation of;^13^ (**a**) the x-component, (**b**) the y-component, (**c**) the z-component, (**d**) the combination of xyz components.

To improve the field uniformity, we add the 1-bit random coding diffusion metasurface on one of the RC walls, facing the antenna (see Fig. 7). Note that the position and direction of the excitation source are independent of the field uniformity. In our case, we place the excitation source in front of the metasurface to reduce the simulation period. Fig. 8 shows the results of standard deviations for the field uniformity in the test zone with the 1-bit random coding diffusion metasurface added to the mechanical stirrer RC. The results indicate that the field uniformity is not significantly improved as compared to the traditional RC case (Fig. 6).

### Numerical modeling of rotating diffusion metasurface loaded in the RC

As noted in the previous section, adding the metasurface does not dramatically improve the field uniformity of the mechanically stirred RC. On the other hand, the presence of the mechanical stirrer severely limits the effective test space of the RC. This issue is especially critical in RCs with limited space. As we know, a RC usually has two stirrer operation modes. One of them is the mode-stirred (so-called stirring mode), where the stirrer, or stirrers, continuously rotate, so that the field changes continuously in the test. Another one is the mode-tuned (so-called step-mode), where the stirrer, or stirrers, rotate step by step, so that the field changes discretely. In the mode-tuned, each stage of the stepped rotation of the stirrer is called on-stirred mode. If we remove the mechanical stirrer and only keep the fixed metasurface in the RC, the fixed metasurface becomes inefficient to improve the field uniformity of the RC. The field in the RC is not sufficiently stirred to ensure a uniform field, and it is not suitable for EMC tests in the RC. For this reason, a rotating diffusion metasurface (metasurfaced RC) is considered accordingly, with an eye towards studying an RC loaded with such rotating metasurface and without the bulky mechanical stirrer.

**Figure 9:**
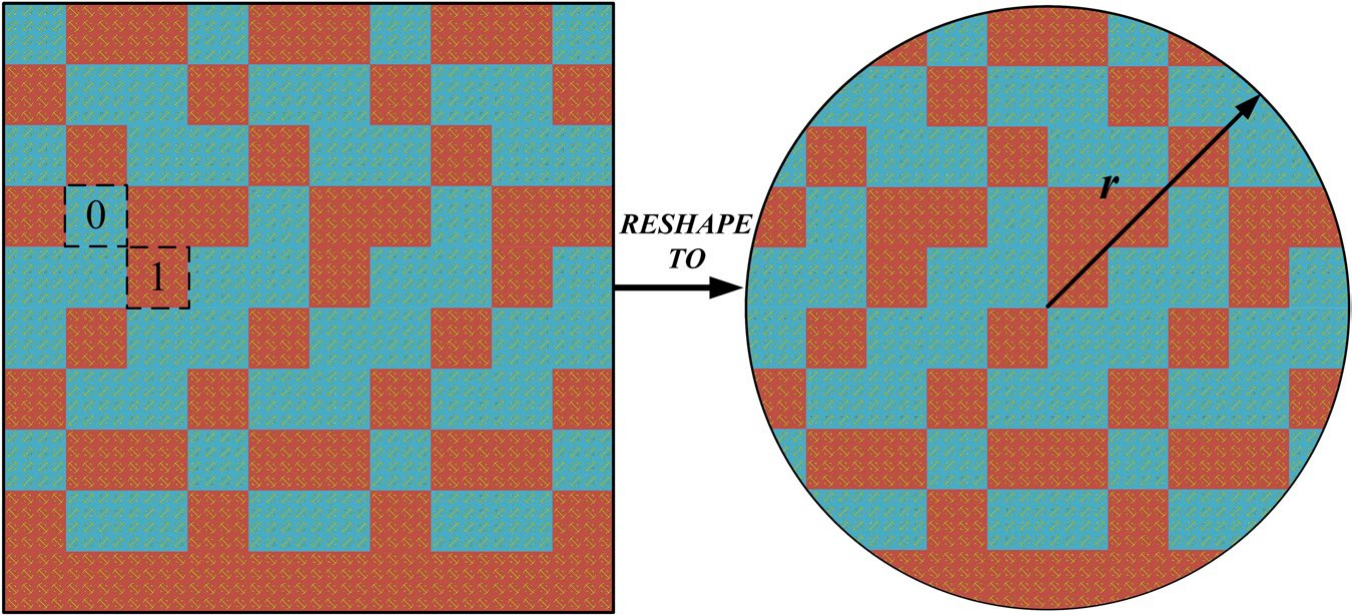
Reshaping of the 1-bit random coding metasurface from a rectangular to circular shape.

**Figure 10:**
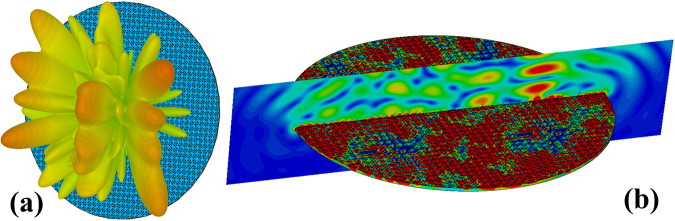
Simulated far-field and near-field patterns of the rotating diffusion metasurface under normal incidence at 1 GHz. (**a**) The far-field scattering pattern. (**b**) The surface current distribution and the near-electric-field distributions of the metasurface.

**Figure 11:**
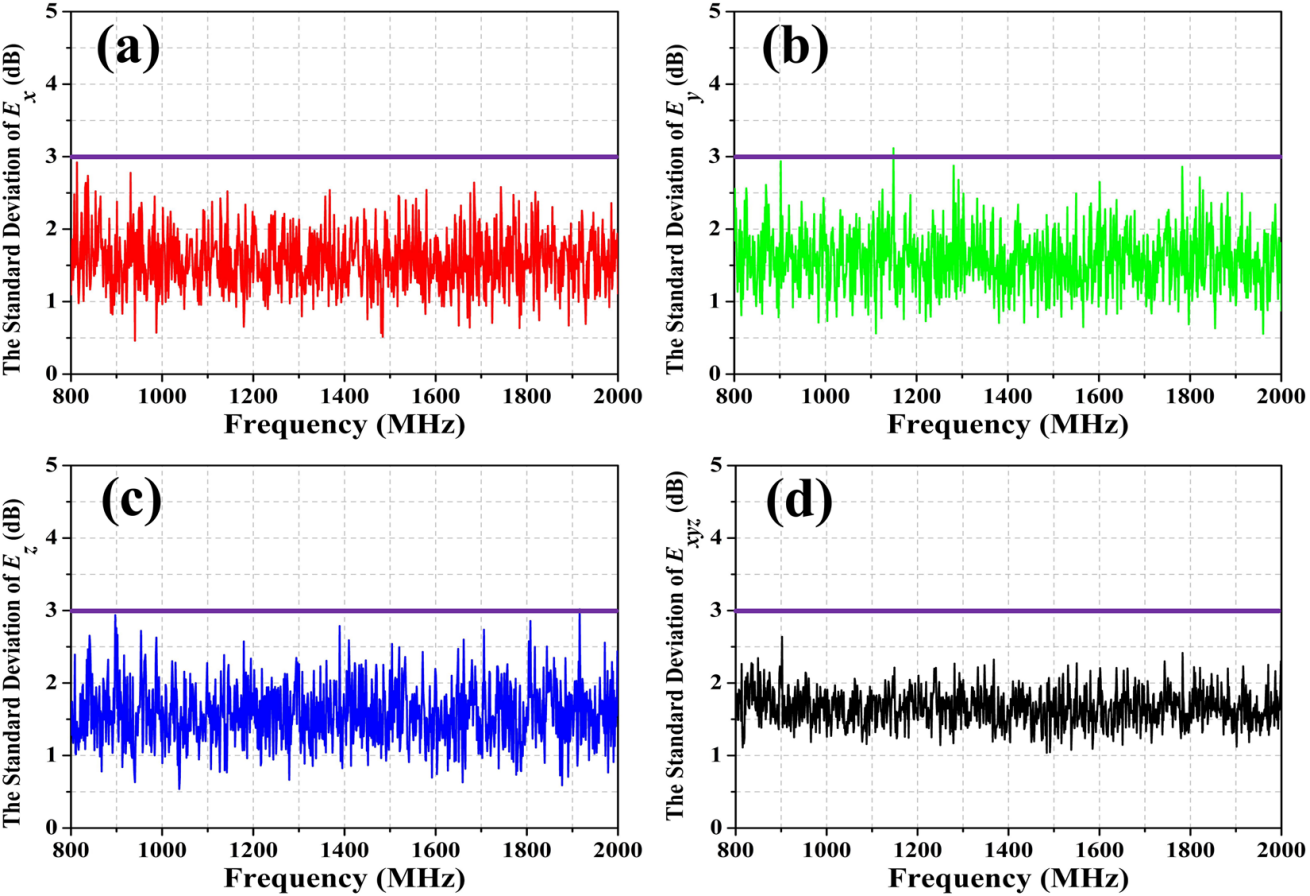
The standard deviations for the field uniformity of the test zone that loaded the rotating 1-bit random coding diffusion metasurface in the RC. The purple lines are the tolerance requirements for the standard deviation of^13^; (**a**) the x-component, (**b**) the y-component, (**c**) the z-component, (**d**) the combination of xyz components.

By reshaping the 1-bit random coding metasurface into a circular form with radius ***r*** = 1.2 m (Fig. 9**)**, 360^o^ rotation in equally sized discrete steps on the wall is possible. Such rotating metasurface acts as a tuner in the RC. To verify the scattering characteristic of the rotating 1-bit random coding metasurface, we simulated the far-field and near-field patterns of the metasurface, as shown in Fig. 10. The far-field pattern shows all-direction backscattering under normal incidence, and the elements demonstrate different resonant states on the metasurface. According to the sampling requirements of^13^, we set the number of tuner steps as 12 for the entire range of operating frequencies from 800 MHz to 2000 MHz and fix the antenna at one position, with coordinates (1.2, 1.25, 1.2). Fig. 11 shows the results of standard deviations for the field uniformity in the test zone loaded with the rotating 1-bit random coding diffusion metasurface in the RC (without mechanical stirrer). All components of the E-field probes meet the requirement of the limits.

**Figure 12:**
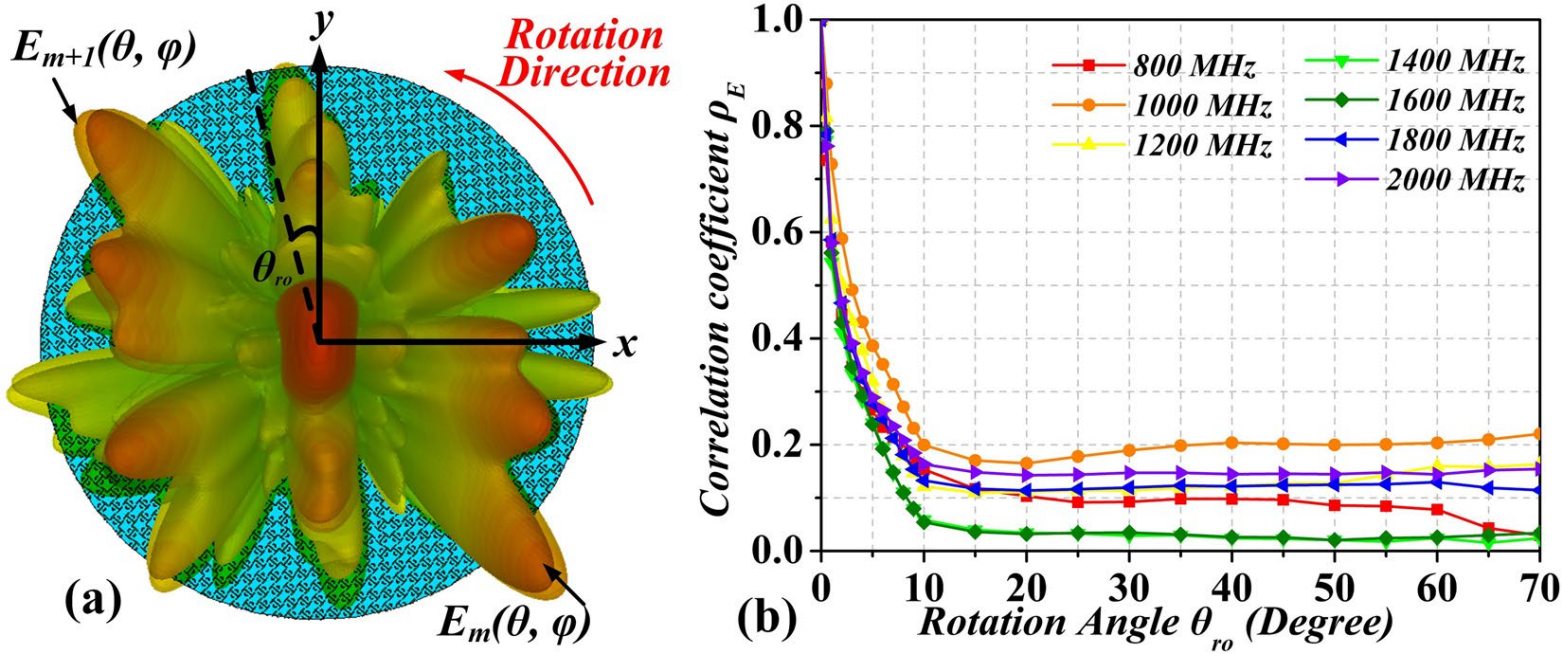
The far-field patterns of the metasurface in different rotation angle ***θ***_***ro***_ (**a**), and the correlation coefficient ***ρ***_***E***_ at different operating frequencies of the RC.

In order to further investigate the stir performance of the RC loaded with the rotating diffusion metasurface, we calculated the correlation coefficient ***ρ***_***E***_ between different three-dimensional far-field patterns of the metasurface corresponding to different rotation angles, as introduced in^32^, i.e.,

7 $${{\boldsymbol{\rho }}}_{{\boldsymbol{E}}}=\frac{|\iint {{\boldsymbol{E}}}_{{\boldsymbol{ref}}}({\boldsymbol{\theta }},{\boldsymbol{\phi }})\cdot {{\boldsymbol{E}}}_{{\boldsymbol{\theta }}\mathrm{ro}}^{\ast }({\boldsymbol{\theta }},{\boldsymbol{\phi }}){\boldsymbol{d}}{\boldsymbol{\Omega }}|}{\sqrt{{\iint |{{\boldsymbol{E}}}_{{\boldsymbol{ref}}}({\boldsymbol{\theta }},{\boldsymbol{\phi }})|}^{2}{\boldsymbol{d}}{\boldsymbol{\Omega }}{\iint |{{\boldsymbol{E}}}_{{\boldsymbol{\theta }}\mathrm{ro}}({\boldsymbol{\theta }},{\boldsymbol{\phi }})|}^{2}{\boldsymbol{d}}{\boldsymbol{\Omega }}}},$$

where (*) means complex conjugate and $\iint \bullet {\boldsymbol{d}}{\rm{\Omega }}={\int }_{0}^{2\pi }{\int }_{0}^{\frac{\pi }{2}}\bullet \,\sin \,{\boldsymbol{\theta }}{\boldsymbol{d}}{\boldsymbol{\theta }}{\boldsymbol{d}}\phi$ is the integral over a unit hemispherical surface. ***E***_***ref***_***(θ, φ)***and ***E***_***θro***_***(θ, φ)*** are the far-field patterns of the rotating metasurface corresponding to the reference angle (0°) and a rotation angle of ***θ***_***ro***_, respectively, as illustrated in Fig. 12 (a). The correlation coefficients with the variations of the rotation angle from ***θ***_***ro***_ = 0° to ***θ***_***ro***_ = 70° in steps of 5° are shown in Fig. 12 (b). From the results, we can observe that all correlation coefficients ***ρ***_***E***_ decrease with the rotation angle in the operating frequency band from 800 MHz to 2000 MHz. This decrease in the correlation coefficient is indicative of the different electric field patterns between the different angles (due to changing boundary conditions), giving rise to an effective improvement of field uniformity.

As discussed in^33–35^ an important parameter to characterize the ability of scattering the diffuse field of an object (and hence to characterize stirrer performance in an RC) is the so-called total scattering cross section (TSCS). Therefore, we have calculated such parameter in the rotating metasurfaced RC (third case study) and in the mechanical stirrer RC (first case study), in order to further compare both cases. The TSCS is given by

8 $${TSCS}=\frac{V}{{\tau }_{s}{c}_{0}},$$

where ***V*** is the volume of the RC, ***c***_***0***_ is the speed of light in free space, and ${\tau }_{s}$ is the scattering damping time of $C(t)$, as defined in^33–35^ InFig. 13, we have plotted $C(t)$ corresponding to the mechanical stirrer and the rotating metasurface. After the estimation of the damping time (${\tau }_{s}$), the TSCS is computed for the two cases see Table 1). In this table, the TSCS value of the rotating metasurface is larger than the one of the mechanical stirrer. As can be seen from the table, the ratio of the occupied space is much larger when the mechanical stirrer is considered (with the significant penalty in the reduction of the test zone in the stirrer RC). Moreover, the average of the field uniformity, given in terms of the averaged standard deviation of the field strengths for the combination of *xyz* components [plots of Figs 6(d) and 11(d)] is 0.25 dB lower for the metasurfaced RC. Hence, the metasurfaced RC has a good stirring performance and can find potential applications in EMC tests in the near future.

**Figure 13:**
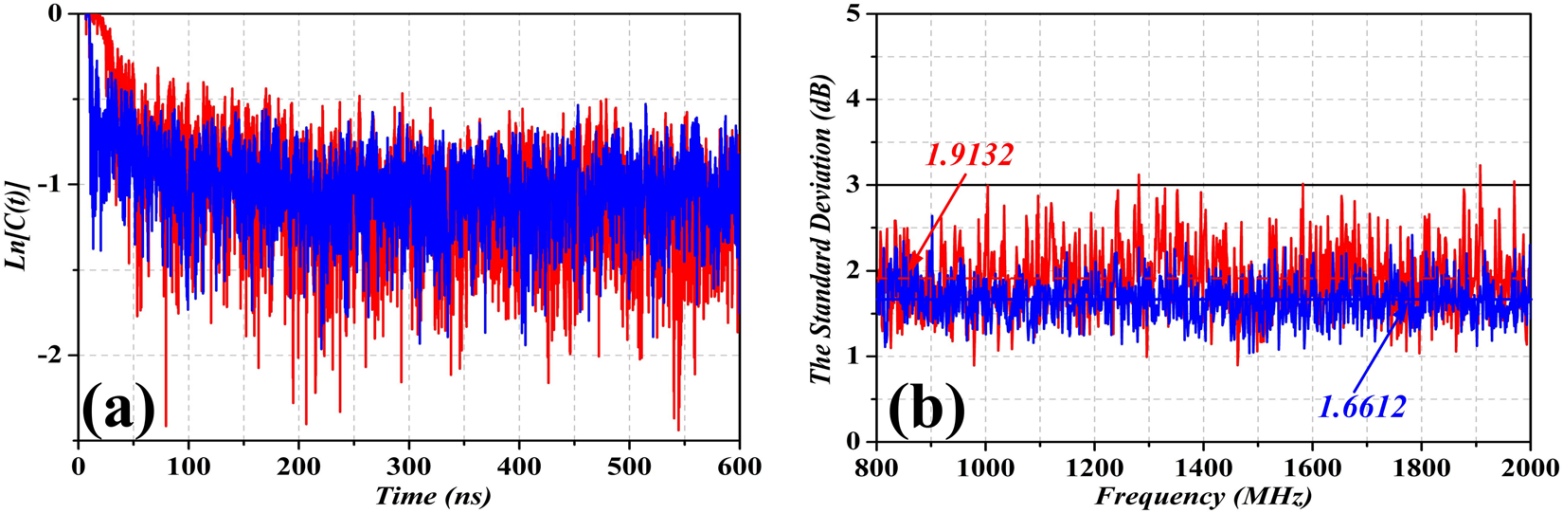
(**a**) Calculated $Ln[C(t)]$ with 30° step degrees, for the mechanical stirrer and for the rotating metasurface, with continuous line in red and blue, respectively. (**b**) Standard deviation with respect to the mean value of maximum measures obtained by each of the 12 steps, for a complete turn of the stirrer (red line is the mechanical stirrer and blue line is the rotating metasurface).

**Table 1:** Comparisons between the RC with mechanical stirrer and RC with rotating metasurface, including TSCS, ratio of the occupied space (volume of stirrer and metasurface over the total volume of the RC), and average standard deviation of the field strengths for the combination of *xyz* components.

	Mechanical Stirrer	Rotating Metasurface
TSCS (m^2^)	1.3874	1.6596
Occupied Space (%)	10.02	0.9
***σ***_***Exyz***_ (dB)	1.9132	1.6612

## Discussion

In this paper, we have analyzed the effects of introducing a coding diffusion metasurface in a RC. To quantify the effect of the loaded metasurface, we have considered three cases for comparison: a (traditional) mechanical stirrer RC (without metasurface), a fixed diffusion metasurface on the wall of the mechanical stirrer RC, and a rotating diffusion metasurface on the wall of the RC (without stirrers). From the results obtained, we can conclude that the field uniformity is only somehow improved for the RC with rotating diffusion metasurface, but due to the lack of mechanical stirrer in this third case, the test zone of the RC can be significantly extended. Note that mechanical stirrers are not subject to bandwidth limitations, different from the metasurfaced RCs. However, bandwidth of the metasurfaced RCs can be extended by using several metasurfaces working in different spectrum. For small size reverberation chambers, the use of the proposed coding diffusion metasurfaces can be of great interest for the optimization of the test zone space, thus allowing for the EMC characterization of large-dimension equipment.
